# Feed additives as frontline tools in mitigating greenhouse gas emissions from agricultural animals

**DOI:** 10.1007/s11250-026-05400-3

**Published:** 2026-10-10

**Authors:** Mona Mohamed Mohamed Yasseen Elghandour, Moyosore Joseph Adegbeye, Abdelfattah Zeidan Mohamed Salem

**Affiliations:** 1 Facultad de Medicina Veterinaria y Zootecnia, Universidad Autónoma del Estado de México, Toluca, Estado de México, C.P. 50000 México; 2 Department of Agriculture, Faculty of Agriculture, University of Africa Toru-orua, Sagbama, Nigeria; 3 Dipartimento di Scienze del Suolo, della Pianta e degli Alimenti (Di.S.S.P.A.), Università degli Studi di Bari Aldo Moro, Via Giovanni Amendola, 165/a, 70126 Bari, BA Italy

**Keywords:** Enteric methane, Feed additives, Greenhouse gas mitigation, Nanoencapsulation, Phytogenics, Ruminant nutrition

## Abstract

Feed additives are among the most promising nutritional strategies for reducing greenhouse gas (GHG) emissions from livestock. Numerous studies have investigated their potential to mitigate GHG emissions from ruminants and pseudo-ruminants using both in vitro and in vivo approaches. These studies have examined a wide range of additives, including phytogenic compounds, microbial additives, seaweeds and microalgae, enzymes, dietary lipids, synthetic inhibitors, agricultural by-products, and nano-encapsulated bioactive compounds. Feed additives have been investigated in various forms, including plant extracts, microbial combinations, nano-encapsulated formulations, and purified bioactive compounds, with several demonstrating promising potential for reducing methane and other GHG emissions while maintaining desirable fermentation characteristics. However, the translation of promising findings into practical livestock production requires further validation through well-designed in vivo studies, particularly to establish effective doses, consistency of responses, and potential interactions with animal species and diets. This editorial highlights recent advances in the use of feed additives for GHG mitigation and discusses key priorities for their practical application. It also recognises the receipt of the 2026 TWAS-UNESCO Award in Agriculture, Nutrition and Food Systems Sciences by Prof. A.Z.M. Salem for his contributions to improving animal production and health and mitigating GHG emissions through innovative feed additives and alternative food resources for cleaner agriculture.

## Introduction

**Fig. 1:**
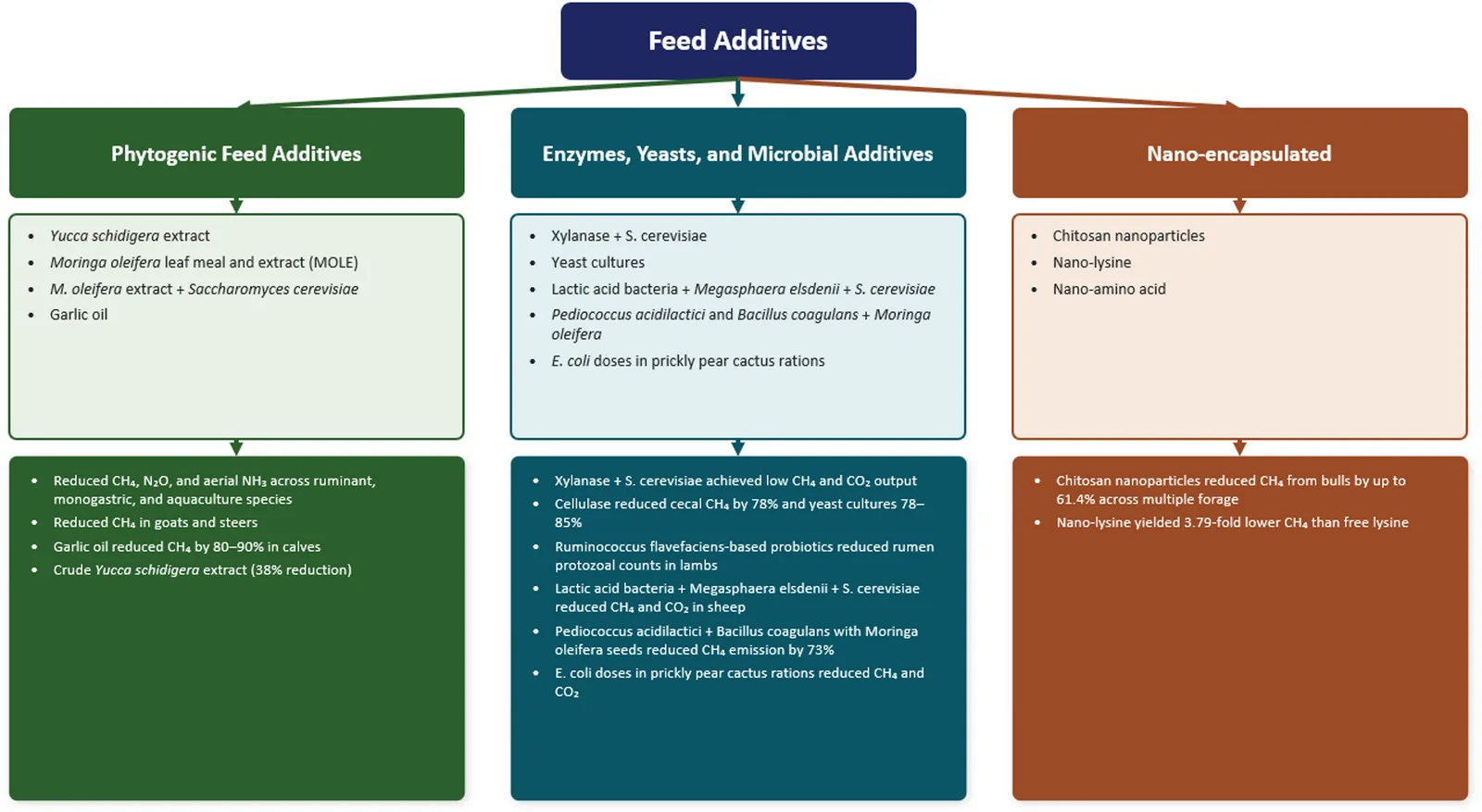
Feed additives used in animal feeding

The livestock sector stands at a critical crossroads, where the problems created through agricultural activities greenhouse gas (GHG) emissions, environmental pollution, amongst others- seem to overshadow its overall positive impact on nutrient provision and nutrient bio-recycling. Ruminants, in particular, are significant contributors to anthropogenic GHG emissions, with enteric fermentation accounting for approximately 30% of agricultural methane emissions (Kebreab et al. 2026). Furthermore, methane (CH₄), Carbon dioxide (CO_2_), and nitrous oxide (N₂O) from manure management and those left on pasture drive these criticisms. The Intergovernmental Panel on Climate Change (IPCC) has consistently highlighted the urgency of reducing these emissions. Without intervention, non-CO₂ GHG emissions from the Global South, primarily from Latin America, Africa, and Asia, are projected to rise by 21–37% by 2050 (Frank et al. 2019). Against this backdrop, the search for cost-effective, biologically sound, and practically deployable strategies to reduce enteric GHG emissions has intensified in recent decades. The scientific community has explored various nutritional strategies, with feed additives emerging as one of the most practical and effective interventions (Hristov 2024). This editorial is occasioned by recognition of Prof. A.Z.M. Salem’s sustained individual contributions to the field, reflected in his receipt of the TWAS-UNESCO 2026 Award in Agriculture, Nutrition, Food Systems Sciences “for improving animal production and health, and mitigating greenhouse gas emissions using innovative feed additives and alternative food resources for cleaner agriculture”. This is an achievement that reflects broader advances the field has seen through the collective contributions of researchers, including the co-authors of this editorial, working on innovative feed additives and greenhouse gas mitigation strategies. It synthesises current evidence on feed additive categories, phytogenics, enzymes, microbial additives, microalgae, dietary lipids, agricultural by-products, and nano-encapsulated compounds, and discuss priorities for translating this science into practice. It draws on a corpus of in vitro and in vivo studies to assess the efficacy of diverse feed additive categories in mitigating GHG emissions from livestock (Fig. 1).

## Phytogenic feed additives

Plant secondary metabolites, tannins, saponins, flavonoids, essential oils, and alkaloids constitute the most extensively studied category of GHG-mitigating feed additives. Tannins suppress methanogenesis through direct antimicrobial and antiprotozoal activity, impaired fibre and protein digestion that reduces fermentation substrate, and direct inhibition of methanogens. Trees, shrubs, and marine phytoplankton will continue to play an important role in ruminant nutrition and GHG mitigation. Condensed tannins from *Caesalpinia coriaria* (cascalote), extracts, and even fruit wastes significantly reduced CH₄, CO₂, and H₂ production in goats and horses. Similarly, at an inclusion level of 3% of the total mixed ration, *C. coriaria* tannins achieved optimal CH_4_ reduction while maintaining acceptable fermentation characteristics (Campos-Pérez et al. 2021; Acosta et al. 2022). Subsequent in vitro work with lambs has corroborated the antimethanogenic potency of cascalote-derived condensed tannins (Hernández Ruiz et al. 2024). Beyond individual trials, a broader meta-analytical evidence confirms this pattern; across 30 experiments and 171 dietary treatments, increasing tannin concentration was linearly related to reduced enteric CH₄ in both in vitro and in vivo settings (Jayanegara et al. 2012), although the magnitude of this effect depends strongly on tannin molecular weight and structural class, with condensed and hydrolysable forms differing in their inhibitory potency (Aboagye and Beauchemin 2019).

Saponins, particularly from *Yucca schidigera* achieve defaunation by disrupting protozoan cell membranes, severing the hydrogen-transfer relationship between protozoa and methanogenic archaea (Xie et al. 2026). This plant demonstrated that it can effectively work as an eco-friendly additive across multiple farm animals. The extract was able to reduce CH₄ by 8.49–69%, N₂O by up to 75%, and aerial NH₃ by 22–100% across ruminant, monogastric, and aquaculture species (Adegbeye et al. 2019). Another plant that has multiple functions, which even goes beyond mitigation is *Moringa oleifera*. It has been extensively studied. Its use in the form of leaf meal and extract (MOLE) demonstrated consistent GHG reductions, while replacing soybean meal with *Moringa* leaf meal reduced CH₄ by 8–10% in goats and steers (Elghandour et al. 2017a), and MOLE combined with soybean hulls as a corn grain substitute delayed CH_4_ initiation and lowered total CH₄ and CO₂ productions (Parra-García et al. 2019). When *M. oleifera* extract was further combined with *Saccharomyces cerevisiae*, proportional CH₄ was reduced by 11.7% at 72 h of incubation, confirming synergistic potential (Pedraza-Hernández et al. 2019). Other notable phytogenics include *Guazuma ulmifolia*, *Crescentia alata*, and garlic oil, the latter achieving 80–90% CH₄ reduction in Holstein dairy calves (Hernandez et al. 2017a). Neem leaf (*Azadirachta indica*) and Indigofera leaf (*Indigofera oblongifolia*) reduced CH₄ emissions while improving rumen fermentation parameters, which was attributed to their rich phenolic profiles (El-Zaiat et al. 2025).

## Exogenous enzymes, yeasts, and microbial additives

Fibrolytic enzymes enhanced cell wall digestibility, shortening rumen retention and limiting fermentation substrate for methanogenesis (Hernandez et al. 2017b). In Holstein calves, a xylanase and *S. cerevisiae* combination achieved the lowest CH₄ production rate and asymptotic CO₂ output, indicating a favourable shift in fermentation end-products (Hernandez et al. 2017b). A fibrolytic enzyme cocktail reduced CH₄ from sugarcane bagasse but increased it from wheat straw at the same dose (Elghandour et al. 2017d), this shows the importance of substrate-specific optimisation. In horses, cellulase reduced cecal CH₄ by 78% in vitro, and yeast cultures achieved reductions of 78–85% (Elghandour et al. 2016). These show that enzyme and yeast additives can be highly effective in pseudo-ruminant hindgut fermenters as well, broadening the applicability of these strategies across livestock species.

Live microbial additives (probiotics) have also demonstrated methane-modulating effects, though results are more variable than for phytogenics or enzymes. A triple combination of lactic acid bacteria (LAB), *Megasphaera elsdenii*, and *S. cerevisiae* reduced proportional CH₄ and CO₂ in sheep (Direkvandi et al. 2021), with LAB stimulating lactate-utilising bacteria that compete with methanogens for Hydrogen (H₂). When *Pediococcus acidilactici* and *Bacillus coagulans* were combined with *M. oleifera* seeds in steers, the rate of CH₄ emission was reduced by 73% (Elghandour et al. 2024). These combinatory approaches are emerging as a particularly promising frontier. In addition, the antagonistic role played by certain microbes can be taken advantage of to reduce the genesis of certain ruminal gases. In Elghandour et al. (2018b), the inclusion of *Escherichia coli* doses in prickly pear cactus rations was able to reduce CH_4_ and CO_2_ production in ruminants.

## Microalgae and dietary lipids

Microalgae rich in eicosapentaenoic acid and docosahexaenoic acid shift ruminal fermentation towards propionate production, providing an alternative H₂ sink that suppresses methanogenesis. Docosahexaenoic acid-enriched microalgae have reduced in vitro CH₄ by up to 80% (Elghandour et al. 2017b). The marine alga *Dunaliella salina* at 3% dry matter (DM) reduced CH₄ across five maize genotypes (Elghandour et al. 2023). Microalgae like *C. vulgaris* was more effective in high-fibre diets than in concentrate-rich diets (Elghandour et al. 2017c), while the combination of sunflower and *Schizochytrium* microalgae at 5% of ration DM significantly decreased both CO₂ and CH₄ production (Elghandour et al. 2017b), providing further confirmation that lipid-rich microalgae constitute anti-methanogenic compounds. Dietary safflower and fish oils combined with *S. cerevisiae*, significantly mitigated CH₄ and CO₂ emissions in horses (Velázquez et al. 2020). In silico analysis further revealed that safflower oil bioactives, particularly acacetin and matairesinol, bind with high affinity to methyl-coenzyme M reductase (MCR), the terminal methanogenesis enzyme, suggesting a direct biochemical inhibition pathway for future drug design (Khusro et al. 2022).

## Synthetic inhibitors and macroalgae

Beyond plant- and microbe-derived compounds, two non-phytogenic strategies have achieved the strongest and most consistent mitigation outcomes reported to date. The small-molecule inhibitor 3-nitrooxypropanol (3-NOP, marketed as Bovaer) specifically inactivates methyl-coenzyme M reductase, the terminal nickel-dependent enzyme common to all methanogenic archaea, by oxidising its Ni(I) cofactor (Duin et al. 2016). Across multiple independent meta-analyses spanning dozens of in vivo trials, 3-NOP supplementation has consistently reduced dairy cattle CH₄ production, yield, and intensity by approximately one-third at recommended doses, with efficacy modulated by dietary NDF and fat content (Kebreab et al. 2023). Separately, the red macroalga *Asparagopsis taxiformis*, owing to its naturally high bromoform content reduced enteric CH₄ yield by over 80% in beef steers when included at low dietary rates (Roque et al. 2021), with dose-dependent reductions of up to 98% reported under feedlot conditions (Kinley et al. 2020; Meo-filho et al. 2024). Together with tannins and 3-NOP, these findings position halogenated and nitro-compound methanogen inhibitors as complementary, mechanistically distinct tools alongside phytogenic and microbial strategies (Beauchemin et al. 2020).

## Nanoencapsulation and agricultural by-products

In ruminant nutrition, one of the challenges associated with additives is the breakdown of the substances in the rumen by microbes or excessive release at the same time. Conventional phytogenic additives such as tannins and saponins can be rapidly degraded in the complex rumen environment before exerting their full anti-methanogenic potential, limiting their in vivo efficacy relative to in vitro assays. Nanoencapsulation offers the prospect of protecting bioactive compounds from premature rumen degradation and enabling targeted delivery. Chitosan nanoparticles reduced CH₄ from bulls by up to 61.4% across multiple forages, outperforming crude *Y. schidigera* extract (38% reduction) in head-to-head comparisons (Botia-Carreño et al. 2024). A complementary study demonstrated that nano-chitosan at a higher dietary protein level (18% CP) effectively reduced CH₄, and Hydrogen sulphide (H₂S) production and improved CH₄ conversion efficiency (Botia-Carreño et al. 2025), confirming that protein level is an important modulator of chitosan nanoparticle efficacy. Among nano-encapsulated amino acids, free methionine produced the least CH₄ owing to its sulphur donation to sulphur-reducing bacteria, creating an alternative H₂ sink, while nano-lysine yielded 3.79-fold lower CH₄ than free lysine (Cayetano De Jesús et al. 2024). These studies collectively indicate that nanoencapsulation can amplify or modulate the anti-methanogenic activity of diverse compounds but requires species- and diet-specific validation before practical deployment.

Agricultural by-products offer a dual benefit of reducing environmental pollution from field burning while modifying rumen fermentation. Soybean hulls, substituted for corn grain at 75–150 g/kg DM, reduced asymptotic CO₂ production in multiple studies (Elghandour et al. 2018a), and their combination with MOLE produced lower CH₄ and CO₂ and improved fermentation pH. Prickly pear cactus as a corn grain substitute shifted fermentation from acetate to propionate, limiting H₂ availability for methanogenesis (Elghandour et al. 2018b). Even in poultry, brown mushroom stem powder at 2–6% replacement of soybean meal produced significant reductions in CO₂, NH₃, and CH₄ without adverse production effects (Abdel-Wareth et al. 2025), extending GHG mitigation science beyond ruminants.

## Research priorities and conclusion

Despite the strength of the in vitro evidence base, translation to practice requires large-scale in vivo validation using direct measurement tools such as GreenFeed, sulfur hexafluoride (SF₆) tracer methodology, or open-circuit respiration chambers especially in the global south. Dose-response relationships must be systematically characterised across species, production stages, and dietary backgrounds, particularly for nano-formulations whose efficacy may be highly context-dependent. Locally available phytogenic resources, such as *Acacia nilotica* and *Ziziphus spina-christi* in Africa (Bekele et al. 2025) and *Pharbitis nil* in Asia (Bharanidharan et al. 2024), also represent potentially low-cost alternatives that may be particularly relevant for smallholder production systems in the Global South.

**Fig. 2:**
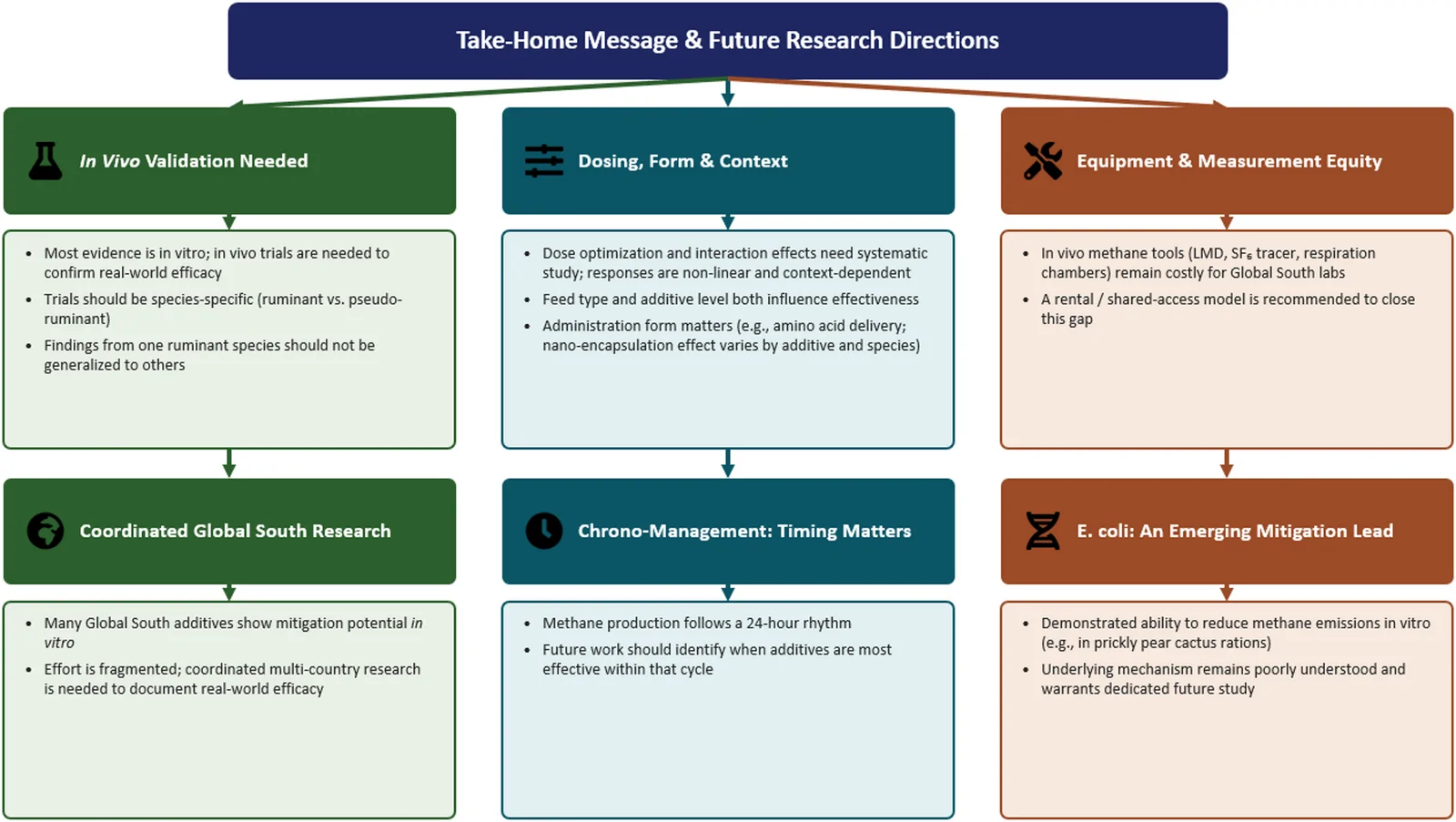
Take-home messages and future research directions for feed additives in greenhouse gas mitigation

Overall, phytogenics, enzymes, probiotics, microalgae, dietary oils, nano-formulations, and functional agricultural by-products offer promising opportunities for reducing enteric CH₄ and other GHG emissions. The next priority is to accelerate rigorous in vivo validation of promising feed additives and establish effective inclusion levels under practical production conditions. These research priorities are summarised in Fig. 2.

Feed additive strategies therefore offer a technically achievable pathway towards lower-emission livestock production, and continued research should focus on translating promising experimental findings into effective, safe, economically feasible, and locally adaptable solutions.

## Data Availability

Not applicable.
